# Seroprevalence of Sparganosis in Rural Communities of Northern Tanzania

**DOI:** 10.4269/ajtmh.16-0211

**Published:** 2016-10-05

**Authors:** Nicholas Kavana, Parthasarathy Sonaimuthu, Christopher Kasanga, Ayub Kassuku, Hesham M. Al-Mekhlafi, Mun Yik Fong, Mohammad Behram Khan, Rohela Mahmud, Yee Ling Lau

**Affiliations:** 1 Department of Veterinary Microbiology and Parasitology, Faculty of Veterinary Medicine, Sokoine University of Agriculture, Morogoro, Tanzania; 2 Tropical Infectious Disease Research and Education Center (TIDREC), Department of Parasitology, Faculty of Medicine, University of Malaya, Kuala Lumpur, Malaysia; 3 Endemic and Tropical Diseases Unit, Medical Research Center, Jazan University, Jazan, Kingdom of Saudi Arabia

## Abstract

In this study, the seroprevalence of sparganosis and its relationship with sociodemographic factors in northern Tanzania have been assessed. A total of 216 serum samples from two rural districts, Monduli and Babati, were tested for sparganosis using an enzyme-linked immunosorbent assay. The seroprevalence of anti-sparganum IgG antibodies was 62.5% (95% confidence interval [CI] = 56.1–68.9) in all age groups. There were significant associations between district (relative risk [RR] = 1.95, 95% CI = 1.42–2.69), education (RR = 1.40, 95% CI = 1.15–1.70), and pet ownership with seropositivity (RR = 1.48, 95% CI = 1.02–2.16) based on univariate analysis. However, only the district was significantly associated with seropositivity (odds ratio = 4.20, 95% CI = 1.89–9.32) in binary logistic regression analysis. Providing health education to people residing in sparganosis-endemic areas is likely to improve the efficacy of preventative measures and reduce human disease burden.

Sparganosis is a human and animal parasitic infection caused by the plerocercoid larvae of diphyllobothroid tapeworms (genus *Spirometra*).^1^ Humans act as accidental hosts whereas dogs, cats, and other mammals serve as the definitive hosts. The first intermediate hosts are copepods, whereas the second intermediate hosts include amphibians and reptiles.^2^ Sparganosis is transmitted to humans by ingestion of water contaminated with infected copepods and ingestion of second intermediate hosts such as frogs or snakes. Sparganosis most often presents clinically as subcutaneous lesions of the chest, abdominal wall, and extremities.^3^ It is difficult to diagnose, and the treatment mostly involves surgical removal of the worms.^4^ Antibody testing is useful to confirm the diagnosis and has also been used in seroepidemiological surveys in susceptible population.^5^ Serodiagnostic tests that target sparganum antigens are good alternatives for sparganosis diagnosis. For instance, enzyme-linked immunosorbent assay (ELISA) is both sensitive and specific for subcutaneous and cerebral sparganosis^6^ and has been used clinically to confirm or exclude cerebral sparganosis.^6^,^7^

There are no comprehensive epidemiological surveys of sparganosis in Tanzania, although cases of sparganosis have been reported in pastoralists (the Maasai) in the Loliondo District of northern Tanzania.^8^ The prevalence of sparganosis in Tanzania is unknown and would be useful for implementing integrated and effective control measures. Furthermore, no serological method has been used to date to diagnose human sparganosis in Tanzania.

In parallel with a sociodemographic survey, serological screening was performed to determine the seroprevalence of sparganosis. A cross-sectional study was carried out in the commonly referred as administrative wards in the rural area of two districts (Babati and Monduli) in northern Tanzania. In Babati District the following wards were involved: Magugu, Mamire, and Galapo. Similarly in Monduli District three wards were involved: Esilalei, Losirwa, and Mto wa Mbu. Sociodemographic data of the inhabitants in the two districts were collected using a pretested questionnaire. A total of 216 serum samples were randomly collected from Babati and Monduli. A written or thumb print consent was acquired after providing an insight on the study. Written permission was obtained from the next of kin or guardian in the case of minors or children. The ethics application was approved by the National Institute for Medical Research, Tanzania (reference no. NIMR/HQ/R.8a/Vol.IX/1285).

An adult cestode representing *Spirometra* was obtained from the small intestine of an infected farm dog at a Minjingu village near Tarangire National Park. The cestode was washed in physiological saline, identified preliminarily to species based on morphological characters and predilection sites. DNA was extracted, and polymerase chain reaction (PCR) was performed to obtain *28S rRNA* gene of *Spirometra* by using the JB10/9 primers.^9^ Crude protein was extracted from the *Spirometra* worm, and the concentration was determined using Bradford assay. Total proteins with a concentration of 20 μg/mL was coated on 96-well microtiter plates (TPP, Trasadingen, Switzerland) and incubated overnight at 4°C. The wells were washed three times with phosphate-buffered saline (PBS) containing 0.1% Tween-20 (PBS-T). Blocking buffer (1% bovine serum albumin in PBS) was added into each well and incubated for 2 hours at 37°C. Patient sera diluted to 1:200 in blocking buffer was added into each well and incubated for 1 hour at 37°C. Peroxidase-labeled goat anti-human IgG (1:2,500 dilutions; KPL Inc., Gaithersburg, MA) was then added followed with 1 hour incubation at 37°C. The wells were incubated with 3,3′,5,5′-tetramethylbenzidine (Amresco, Solon, OH) for 30 minutes in dark. The reaction was stopped by adding 2NH_2_SO_4_, and the optical density (OD) was read at 450 nm. All samples were run in duplicates. The cutoff value was calculated as the *M*_N_ + 2σ of the healthy donor sera group, where *M*_N_ and σ are the mean OD and the standard deviation, respectively. Samples with OD values higher than *M*_N_ + 2σ were considered positive. The SPSS v.20 (SPSS IBM, New York, NY) was used to conduct the statistical analysis. Univariate and multivariate tests were performed to test the association, and the level of significance was placed at *P* ≤ 0.05 for each analysis.

The adult cestode was confirmed to be *Spirometra erinacei* through PCR and phylogenetic analysis. The nucleotide sequence identity was 100% similar to GenBank *S. erinacei 28S rRNA* gene (accession no. AF004717.2). Among the 216 participants in this study, 135 (62.5%, 95% CI = 56.1–68.9) were positive for sparganosis. Univariate analysis was performed to check the association of positive results with gender, age group, district, education, pets, and water consumption (Table 1). All risk factors contributed to the seropositivity except for gender (relative risk [RR] = 0.889, 95% confidence interval [CI] = 0.696–1.14) and age group (RR = 0.851, 95% CI = 0.669–1.08). Binary logistic regression indicated that the district was the most important factor in terms of sparganosis infection (Table 2). Overall, 100% of the participants had no prior knowledge about sparganosis.

Our survey results are consistent with findings from a study from South Korea, in which diagnosed patients were unaware of sparganosis^6^; in far eastern populations, the seroprevalence of sparganosis has been reported to be between 1.6% and 3%.^10^–^12^ Serum ELISA has been shown to be a reliable method for the preoperative diagnosis of sparganosis.^13^ It has been long hypothesized that human sparganosis is a common infection in east Africa,^8^,^14^ and this study provides further evidence for the claim. Molecular characterization of seven of the 37 human sparganosis specimens from Ethiopia and South Sudan showed high similarity to *Spirometra erinaceieuropaei*.^15^

Sparganosis can be transmitted to humans via unboiled water containing procercoid-infected *Cyclops*. In this study, the majority of participants (94.9%) consumed unboiled water from running springs and rivers. Univariate analysis of the data showed that there was a higher risk (RR = 2.36, 95% CI = 0.895–6.23) of sparganosis in individuals drinking contaminated water. This finding is consistent with a study from South Korea reporting sparganosis infection arising in people drinking from contaminated springs.^16^ However, the sample size for individuals who drink boiled water is extremely small, which makes the statistical analysis quite biased. These data are noteworthy as they depict the proportion of the population that has a lack of access to clean drinking water. In Masailand, the majority of the infections were confined to the ankle area, suggesting that the hosts acquired the infection by standing in water bodies contaminated with *Cyclops*.^8^

Dogs and cats are definitive *Spirometra* hosts: the adult worms produce eggs in the small intestines of dogs and cats, which are dispersed during defecation. Eggs develop and hatch into coracidia when they make contact with water, which are then ingested and develop into infective procercoids in body cavities. In the present study, a higher infection rate was observed (RR = 1.48, 95% CI = 1.02–2.16) in participants with dogs and cats; these pets usually roam free outside the residential area. No differences in seropositivity were observed between males and females. In rural Tanzania, men are actively involved in animal keeping and farming, unlike women, who tend to have domestic roles. Men might therefore be expected to be more prone to sparganosis; however, the relatively low numbers of male participants may have masked this finding. Individuals without formal education were more likely to be infected (RR = 1.40, 95% CI = 1.15–1.70); education is known to play an important role in health-promoting behavior, thereby reducing infection in educated individuals. In terms of the districts studied, Monduli residents were more susceptible to infection (RR = 1.95, 95% CI = 1.42–2.69), although the smaller sample size in the Babati District may explain this phenomenon. Furthermore, the Monduli District (OR = 4.20, 95% CI = 1.89–9.32) remained the most important factor associated with seropositivity in binary logistic regression.

Crude adult *S. erinacei* extracts have previously been used in the serodiagnosis of sparganosis, with both the sensitivity and specificity being higher than 90%.^17^ Furthermore, crude saline extracts of sparganum larvae also demonstrated high sensitivity (> 85%) and specificity (> 95%) toward detection of human sparganosis,^6^ indicating that the results obtained in the present study are reliable. The cross-reactivity of serum samples with other helminthiases, such as clonorchiasis,^17^ cysticercosis,^18^ paragonimiasis,^19^ and *Taenia saginata*^6^ could be due to a previously acquired infection.

In conclusion, the inhabitants of the Babati and Monduli districts of northern Tanzania lack knowledge and have poor practices with respect to sparganosis infection. Serological testing revealed that sparganosis is highly prevalent in inhabitants of these two districts, with Monduli having a higher seroprevalence than Babati. Therefore, there is a dire need for health education programs and community mobilization to enhance prevention and improve knowledge about sparganosis transmission in these communities.

## Figures and Tables

**Table 1:** Univariate analysis for the association between risk factors and seroprevalence

Risk factor	*N*/total (%)	RR (95% CI)	*P* value
Gender			
Male	35/61 (57.4)	0.889 (0.696–1.14)	0.329
Female	100/155 (64.5)	–	–
District			
Monduli	116/168 (69.0)	1.95 (1.42–2.69)	0.000
Babati	19/48 (39.6)	–	–
Age group			
≤ 50	110/181 (60.3)	0.851 (0.669–1.08)	0.233
> 50	25/35 (71.4)	–	–
Education			
Uneducated	59/77 (76.6)	1.40 (1.15–1.70)	0.001
Educated	76/139 (54.7)	–	–
Pets			
Yes	65/92 (70.7)	1.48 (1.02–2.16)	0.033
No	70/124 (56.5)	–	–
Boiled water			
Yes	3/11 (27.3)	2.36 (0.895–6.23)	0.013
No	132/205 (64.4)	–	–

CI = confidence interval; RR = relative risk.

**Table 2:** Multivariate analysis for the association between risk factors and seroprevalence

Risk factor	aOR (95% CI)	*P* value
Gender		
Male/female	0.703 (0.345–1.44)	0.334
District		
Monduli/Babati	4.20 (1.89–9.32)	0.000
Age group		
≤ 50/> 50	0.67 (0.217–2.07)	0.486
Education		
Uneducated/educated	2.32 (0.979–5.50)	0.560
Pets		
Yes/o	1.34 (0.684–2.62)	0.394
Boiled water		
Yes/no	3.49 (0.845–14.4)	0.084

aOR = adjusted o0064ds ratio; CI = confidence interval.

## References

[1] Li MW, Song HQ, Li C, Lin HY, Xie WT, Lin RQ, Zhu XQ (2011). Sparganosis in mainland China. Int J Infect Dis.

[2] Zhao YM, Zhang HC, Li ZR, Zhang HY (2014). Scrotal sparganosis mimicking scrotal teratoma in an infant: a case report and literature review. Korean J Parasitol.

[3] Cho JH, Lee KB, Yong TS, Kim BS, Park HB, Ryu KN, Park JM, Lee SY, Suh JS (2000). Subcutaneous and musculoskeletal sparganosis: imaging characteristics and pathologic correlation. Skeletal Radiol.

[4] Chang KH, Cho SY, Chi JG, Kim WS, Han MC, Kim CW, Myung H, Choi KS (1987). Cerebral sparganosis: CT characteristics. Radiology.

[5] Park HY, Lee SU, Kim SH, Lee PC, Huh S, Yang YS, Kong Y (2001). Epidemiological significance of sero-positive inhabitants against sparganum in Kangwon-do, Korea. Yonsei Med J.

[6] Kim H, Kim SI, Cho SY (1984). Serological diagnosis of human sparganosis by means of micro-ELISA. Korean J Parasitol.

[7] Moon WK, Chang KH, Cho SY, Han MH, Cha SH, Chi JG, Han MC (1993). Cerebral sparganosis: MR imaging versus CT features. Radiology.

[8] Schmid H, Watschinger H (1972). Sparganosis in the Masailand. Acta Trop.

[9] Lee SU, Huh S, Phares CK (1997). Genetic comparison between *Spirometra erinacei* and *S. mansonoides* using PCR-RFLP analysis. Korean J Parasitol.

[10] Kong Y, Cho SY, Kang WS (1994). Sparganum infections in normal adult population and epileptic patients in Korea: a seroepidemiologic observation. Korean J Parasitol.

[11] Lee KJ, Bae YT, Kim DH, Deung YK, Ryang YS (2002). A seroepidemiologic survey for human sparganosis in Gangweon-do. Korean J Parasitol.

[12] Lee SH, Kim MN, Back BY, Choi JY, Kim TH, Hwang YS (2003). Analysis of parasite-specific-antibody positive patients for *Clonorchis sinensis*, *Paragonimus westermani*, cysticercus and sparganum using ELISA. Korean J Lab Med.

[13] Ou Q, Li SJ, Cheng XJ (2010). Cerebral sparganosis: a case report. Biosci Trends.

[14] Nelson GS, Pester FR, Rickman R (1965). The significance of wild animals in the transmission of cestodes of medical importance in Kenya. Trans R Soc Trop Med Hyg.

[15] Eberhard ML, Thiele EA, Yembo GE, Yibi MS, Cama VA, Ruiz-Tiben E (2015). Case report: thirty-seven human cases of sparganosis from Ethiopia and South Sudan caused by *Spirometra* spp. Am J Trop Med Hyg.

[16] Lee SH, Chai JY, Seo BS, Cho SY (1984). Two cases of human infection by adult of *Spirometra erinacei*. Korean J Parasitol.

[17] Yang HJ, Kong Y, Lee SU, Huh S (1998). Applicability of crude extracts of adult *Spirometra erinacei* for serodiagnosis of sparganosis. P Helm Soc Wash.

[18] Nishiyama T, Ide T, Himes SR, Ishizaka S, Araki T (1994). Immunodiagnosis of human sparganosis mansoni by micro-chemiluminescence enzyme-linked immunosorbent-assay. Trans R Soc Trop Med Hyg.

[19] Yamasaki H, Nakamura T, Intapan PM, Maleewong W, Morishima Y, Sugiyama H, Matsuoka H, Kobayashi K, Takayama K, Kobayashi Y (2014). Development of a rapid diagnostic kit that uses an immunochromatographic device to detect antibodies in human sparganosis. Clin Vaccine Immunol.

